# The Effects of Ultrasonic Scaling and Air-Abrasive Powders on the Decontamination of 9 Implant-Abutment Surfaces: Scanning Electron Analysis and In Vitro Study

**DOI:** 10.3390/dj10030036

**Published:** 2022-03-01

**Authors:** Francesco Gianfreda, Patrizio Bollero, Maurizio Muzzi, Andrea Di Giulio, Eleonora Nicolai, Luigi Canullo

**Affiliations:** 1 Department of Industrial Engineering, University of Rome “Tor Vergata”, 00133 Rome, Italy; francesco.gianfreda@ptvonline.it; 2 Department of System Medicine, University of Rome “Tor Vergata”, 00133 Rome, Italy; patrizio.bollero@ptvonline.it; 3 Department of Science, University Roma Tre, Viale G. Marconi, 446, 00146 Rome, Italy; maurizio.muzzi@uniroma3.it (M.M.); andrea.digiulio@uniroma3.it (A.D.G.); 4 Department of Experimental Medicine, University of Rome Tor Vergata, Via Montpellier 1, 00133 Rome, Italy; nicolai@med.uniroma2.it; 5 Independent Researcher, 00198 Rome, Italy

**Keywords:** SEM, in vitro study, abutment surface, abutment connection, peri-implant response, air-abrasive powders, ultrasonic implant scaling, implant decontamination

## Abstract

(1) Background: The aim of this study is to understand from a microscopic point of view whether bicarbonate air-abrasive powders associated with ultrasonic instruments can decontaminate nine different surfaces used for the abutment/implant junction. Fibroblast growth was carried out on decontaminated surface in order to understand if there are significative differences in terms of biocompatibility. (2) Methods: After taking samples of patient plaque, nine different surfaces were contaminated and analyzed by SEM, then their wettability was evaluated. Fibroblasts were cultured on the decontaminated surfaces to understand their ability to establish a connective tissue seal after decontamination. The results were analyzed from a statistical point of view to hypothesize a mathematical model capable of explaining the properties of the surfaces. (3) Results: A negative correlation between roughness and contamination has been demonstrated, whereas a weak correlation was observed between wettability and decontamination capacity. All surfaces were topographically damaged after the decontamination treatment. Grade 5 titanium surfaces appear tougher, whereas anodized surfaces tend to lose the anodizing layer. (4) Conclusions: further studies will be needed to fully understand how these decontaminated surfaces affect the adhesion, proliferation and differentiation of fibroblasts and osteoblasts.

## 1. Introduction

One of the most investigated aspects in contemporary dentistry is the long-term maintenance of dental implants and the prevention of peri-implantitis. Peri-implantitis has recently been defined by Schwartz et al. [1] as a pathological condition affecting the tissues around the implants. This condition is characterized by inflammation of the peri-implant connective tissue and by a non-linear, accelerated bone loss supporting pattern [1]. One of the most important aspects concerning the 2017 World Workshop on the Classification [1] of Periodontal and Peri-Implant Diseases and Conditions is certainly the clinical definition of peri-implantitis, described as a larger probing depth than the baseline associated with clinical signs of inflammation.

When no previous probing and radiographs are present, Berglundh et al. [2] suggested defining peri-implantitis as bone levels ≥ 3 mm apical of the most coronal portion of the intra-osseous part of the implant together with bleeding on probing.

In fact, since there is no official definition, many author tried to define peri-implantitis by setting different thresholds. Consequently, this led to a lack of homogeneity also in the epidemiological data. A study by Derks et al. [3] reported that 45% of implanted patients are affected by peri-implantitis. Atieh [4] reported an 18.8% prevalence in patients demonstrating data in agreement with a review of Rakic et al. [5] (18.5% of patients with implants). Several factors can influence this susceptibility to peri-implantitis [6,7], increased by poor patient compliance, such as poor oral hygiene and smoking habits. Furthermore, uncontrolled diabetes, periodontitis history and biphosphonates are factors that can lead to an altered immune response. Shifting out attention from the host to the implant-supported restoration, it is possible to observe different aspects that are not present in natural teeth. The International Brainstorming Meeting on etiologic and risk factors of Periimplantitis in 2014 [8] pointed out that peri-implantitis is not triggered exclusively by plaque-associated injuries. Other factors are responsible for bone reconstruction failure, such as incorrect 3D implant positioning, incorrect soft tissue management, abutment unscrewing, or overload induced implant fracture, incorrect finishing line positioning with soft tissue margin line, infection of internal tolerance spaces at the connection and presence of non-resorbable cement. All the implants have a macro- and micro-geography of surface that does not make the biofilm decontamination easy [9]. In addition, the implant/abutment junction has a mismatch that favors the accumulation of bacteria within the implant connection [7]. In less-stable prosthetic connections, this causes a pumping effect of the junction which leads to the accumulation and release of bacteria [10]. For this reason, bacterial colonization of the connection inner portion can be associated with peri-implant bone loss and peri-implantitis. From a clinical point of view, in case of peri-implantitis and mucositis, it would, therefore, be desirable not to simply debride the connection with ultrasound and airflow, but remove and decontaminate the prosthetic connection and abutment [11,12]. Many authors have suggested how micro-geographically smoother surfaces can promote decontamination from biofilm using ultrasound and air polishing [13,14,15]. For many clinicians, the treatment of mucositis and peri-implantitis is a challenge that often does not give the desired results. In fact, the surface micro-topography can influence the biofilm in terms of bacteria adhesion and release of pro-inflammatory and necrotizing cytokines [16]. It can often result in the formation of ecological niches that are difficult to decontaminate. Many authors have proposed the use of powders and ultrasounds for biofilm removal [14,15,17].

Often this practice can result in incomplete removal of the biofilm, which can be responsible for incomplete healing. In fact, in the deeper pockets the action of ultrasound loses its effectiveness [18], whereas the airflow can lead to the formation of emphysema [19]. From a macroscopic point of view, the use of currettes cannot guarantee the cleansing of surface micro-topographies.

Some authors have investigated the ability of different air powder abrasive systems to decontaminate different surfaces. A study by Wei et al. [20] demonstrated that bicarbonate powders have a more effective action even at lower pressure than glycine powder. Another in vitro study compared the efficacy of sodium bicarbonate, glycine and erythritol powders and demonstrated better results with the use of the larger particle size powder [13].

It is interesting to note that the use of lasers can be a supportive modality for conventional non-surgical treatment. The use of different types of lasers seems to obtain promising and similar results with the presence of fewer bacteria after treatment. However, it is also necessary to pay attention to the risk of creating tissue damage due to overheating caused by the laser [21,22].

In this study, attention was focused on the use of ultrasound and bi-carbonate powders because, among general dentists, they are common materials in a dental office.

The purpose of the in vitro study is to understand from a microscopic point of view whether the use of bicarbonate powders associated with ultrasonic instruments can decontaminate nine different kinds of surface used for the manufacturing of abutment/implant junction. Furthermore, attention will be paid to the regrowth of fibroblasts above the decontaminated surfaces in order to understand if there are significative differences in terms of biocompatibility of the different substrates.

## 2. Materials and Methods

### 2.1. Sample Preparation

Nine different types of sterile and decontaminated surfaces (Sweden and Martina, Padua, Italy) were used for this study:

- Titanium Grade 4 Surfaces: RS: machined surface; RS-GI: machined Anodized surface; UTM: “micro-grooved” Ultrathin Threaded Microsurface; UTM-GI: “micro-grooved” Anodized Ultrathin Threaded Microsurface; XA: “micro-grooved” Thin Machined surface; XA-GI: Anodized “micro-grooved” Thin Machined surface; ZT: zirconium oxide-sandblasted and mineral acids-etched surface.
- Titanium machined Grade 5 Surface: RS-GR5.
- Yttria-stabilized zirconia ceramic surface: ZR.

All disks had a diameter of 10 mm and a height of 3 mm. After manufacturing, all the titanium discs underwent the same standard cleaning and sterilization procedure that is used for commercial dental implants.

### 2.2. Sample Size

In total, 3 discs per surface type underwent surface and micro-topography analyses, 3 discs per surface type underwent wettability analysis and 3 discs per surface underwent roughness analysis. A number of 18 discs for each surface were incubated to be contaminated with plaque from the oral cavity. Therefore, in total, 162 discs were contaminated. Among these 12 disks per surface, for a total of 108 discs, were decontaminated with powders and ultrasounds.

Finally, primary fibroblast-like HDFn cells (Human Dermal Fibroblasts, neonatal cells—Thermo Fisher Scientific, Waltham, MA, USA) were cultured on 6 sterile and decontaminated discs of each surface in DMEM (Dulbecco’s Modified Eagle’s Medium Gibco). The test-group concerning the growth of fibroblasts was made up of half of the disks decontaminated with powders and ultrasounds (54 disks).

### 2.3. Contact Angle Characterization

Wettability of the disc samples was estimated by measuring the contact angle with water. A drop of distilled deionised water, with a volume of 3 µL, was gently poured with a micropipette on the tested surfaces, and one minute after its deposition the resulting sessile drop was photographed using an EC3 ccd camera (Leica Microsystems, Wetzlar, Germany) coupled to a Navitar zoom 6000 macro lenses (Navitar, New York, NY, USA). In order to test different areas of the sample and to reduce the risk of surface alteration by water, each disc was measured at three different spots.

### 2.4. Biofilm Cultivation

Subgingival plaque was collected from hopeless elements extracted for periodontal reasons and with probing greater than 7 mm. Plaque was incubated in 2 mL of LB Broth (Lennox L-broth base, Invitrogen, MA, USA), a generic culture medium for bacteria, for 24 h at 37 °C in oscillating incubator to obtain the inoculum for biofilm cultivation. Discs were placed into wells of 6-well cell plates (Corning Life Sciences, Woburn, MA, USA), covered with 3 mL of subgingival human plaque suspension + 2 mL of fresh LB broth medium. Bacteria and discs finally were incubated for 7 days, at 37 °C. The LB medium was replaced every 24 h. After 7 days of cultivation, the medium rich in bacteria was removed, and the biofilm-covered discs were transferred into new wells of a sterile 6-well plate. All discs were washed with PBS 1X, then discs used for Cell culture were leaved in PBS 1X, whereas discs used for microscopy were fixed in formalin solution neutral buffered 10% (Sigma Aldrich, MO, USA) for 15 min at room temperature and dehydrated using solutions with an increased concentration of alcohol (50%, 5 min—75%, 5 min—80%, 5 min, 95%, 5 min and 100%, 30 min). Fixed and non-fixed discs were stored at 4 °C.

### 2.5. Ultrasonic Debridement and Air Polishing

The Perio-Flow nozzle (AIR-FLOW Master Piezon; EMS, Nyon, Switzerland) was directed to the nine types of implant/abutment surfaces with an angle of 60–90 degrees. Each surface was debrided for 30 s with bicarbonate powder with the dimension of 40 µm (AIR-FLOW Powder Supragengival; EMS) for two times, before and after the ultrasonic debridement. An ultrasonic device (AIR-FLOW Master Piezon; EMS) with an EMS PS Ultrasonic Tip of stainless steel (EMS, Nyon, Switzerland) under maximum irrigation and 80% power was used to debride the titanium disks for one minute after the first treatment with powder.

### 2.6. Fibroblast Cultivation after Biofilm Removal

For test preparation, fibroblast-like HDFn cells (Human Dermal Fibroblasts, neonatal cells—Thermo Fisher Scientific, Waltham, MA, USA) were cultured in DMEM (Dulbecco’s Modified Eagle’s Medium Gibco), supplemented with 2 mM L-glutamine, 1% *v/v* pen/strep, 1000 mg/L glucose (Thermo Fisher Scientific, Waltham, MA, USA) with 10% FBS (fetal bovine serum; Gibco, Thermo Fisher Scientific, Waltham, MA, USA) without any antibiotics, in tissue culture flasks (TPP, Trasadingen, Switzerland). Cells were cultured at 37 °C in a humidified atmosphere with 5% CO_2_ and split at 80% of confluence by trypsin/EDTA (PAA Laboratories, Cölbe, Germany) to attain an adequate number of cells for the test and further microscopic analysis of the cell surface covering. Cells were used within 10 passages (from origin). After biofilm removal treatment, the cells were seeded onto the top of the discs at the density of 190 cells per mm^2^ and were cultured at 37 °C in a humidified atmosphere with 5% CO_2_ for 5 days. After incubation, the samples were fixed with 2.5% glutaraldehyde in phosphate-buffered saline (PBS) and stored at 4 °C until further processing. Samples were washed three times with PBS for 5 min each, washed two times in deionised water for 5 min each and then dehydrated in a graded series of aqueous ethanol solutions (10%, 30%, 50%, 70%, 90%) and in 100% ethanol on ice for 15 min for each step. The samples were then allowed to reach room temperature before the ethanol was replaced with new 100% ethanol at room temperature for 10 min.

### 2.7. Topographic Analysis

Samples were observed using a Gemini 300 field emission SEM (Carl Zeiss AG, Jena, Germany), at the electron microscopy laboratory of Roma Tre University (LIME, Rome, Italy); all micrographs were acquired by detecting secondary electrons and using an accelerating voltage set at 5.0 kV. Representative samples of the nine different disc types were directly placed on stubs using a double-sided carbon adhesive disc and examined by SEM. Discs obtained after fibroblast cell culturing and those used for evaluating bacterial biofilm growth were chemically fixed, as described in the respective sections, and subsequently dehydrated in a graded ethanol series. Samples for the biofilm assessment were air dried in a fume hood, whereas discs with fibroblasts were critical point dried in a CPD 030 unit (BalTec, Balzers, Liechtenstein). Prior to SEM analyses, dehydrated samples were secured to a stub with a double-sided carbon adhesive disc and coated with a thin layer of gold (approximately 30 nm) using a K550 sputter coater (Emithech, Kent, UK).

A stereoscopy of images obtained by setting the angle of inclination allowed the processing of the results through specific software (Mex 6.0, Alicona Imaging, Chicago, IL, USA).

The three-dimensional images obtained made it possible to calculate the arithmetical mean height (Sa). In this analysis, the images were obtained with a 2000× magnification. The roughness parameters according to ISO25178 were obtained from reconstructed images of an area of 80 × 110 μm.

### 2.8. Disk Analysis after Biofilm Removal and Fibroblast Growth

For each disk of the “contaminated” and “decontaminated” group, 6 micrographs were taken randomly with a 4000× magnification.

In the 108 discs on which the fibroblast growth was carried out (54 Test and 54 Control), 6 micrographs were taken with 1000× magnification in order to better observe the distribution of the fibroblasts.

**Figure 1:**
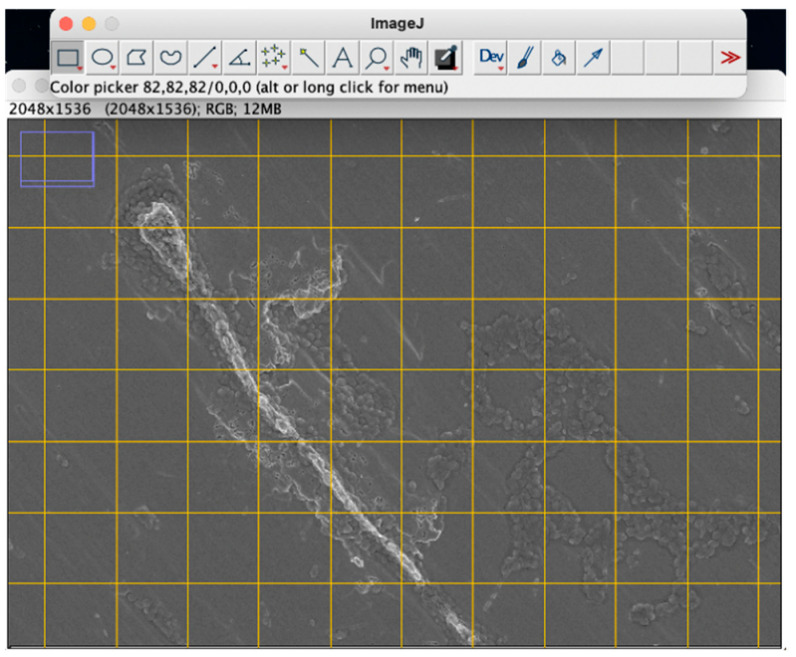
Example of a micrograph processed with ImageJ with plugin “Grid overlay”.

All surfaces were randomized blinded and independently analyzed by two microscopy experts with ImageJ (v1.50, US National Institutes of Health, Bethesda, MD, USA) and the “Grid Overlay” plugin which created a grid with cross points 11 × 7 (Figure 1). At each crossing point, it was assessed whether microbes were present in the contaminated and decontaminated surfaces. In the surfaces where the growth of fibroblasts had been carried out, the presence of cells at the crossing points was assessed. The results for each grid have been reported as a percentage to allow a homogeneous statistical survey.

### 2.9. Statistical Analysis

Statistical analysis was performed using Mathlab (Mathworks, Inc., Natick, MA, USA). Specifically, linear and non-linear regressions were calculated between the different groups. The *t*-tests were performed to evaluate the significance between the samples through the *p*-value.

## 3. Results

### 3.1. Contact Angle Characterization

Contact angle analysis revealed different values which were summarized in Table 1. Analysis of variance showed that the differences among the disc groups were significant (*p* = 0.01) and analysis of the data highlighted the presence of differences among the various groups of discs (*p* value 0.05). The analysis of the data highlighted that there are significant differences between the considered group of discs and the other groups, with the following exceptions reported below. Wettability of UTM discs showed no significant differences with that of XA discs; GR5 contact angles showed no meaningful differences with those of XA GI and RS GI discs, whereas RS Discs showed no significant differences with UTM GI discs. 

### 3.2. Roughness

Arithmetical mean height (Sa) of the surfaces was calculated to validate the roughness of the surfaces. All results are summarized in Table 1. The anodizing treatment of the RS (Sa 0.20), UTM (Sa 0.60) and XA (Sa 21.00) surfaces showed no differences in terms of micro-roughness. The use of a grade 5 titanium compared with a grade 4 titanium did not lead to differences in roughness between RS and RS-GR5. However, these surfaces are extremely smooth (Sa 0.2); therefore, speculations could be made about this result.

### 3.3. SEM Analysis

**Figure 2:**
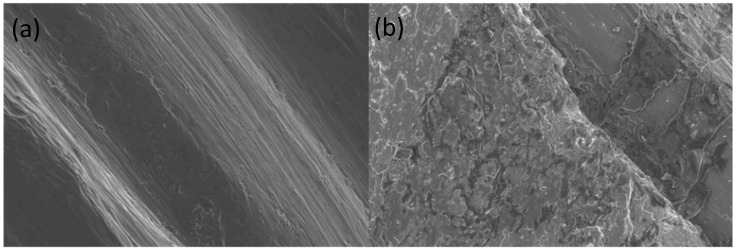
UTM-GI surfaces before (**a**) and after (**b**) the treatment for the decontamination. It is possible to observe the almost complete loss of anodization after the treatment with powder and ultrasonic scaling (**b**).

**Figure 3:**
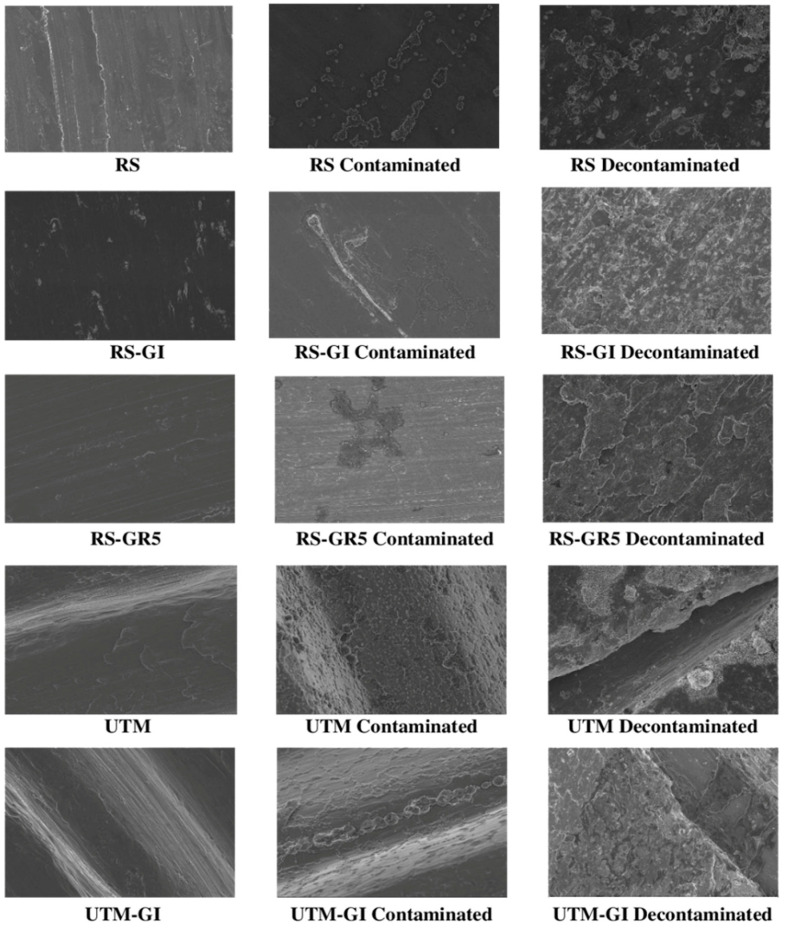
Micrographs of the sterile, contaminated by plaque and decontaminated RS, RS-GI, RS-GR5, UTM and UTM-GI surfaces.

**Figure 4:**
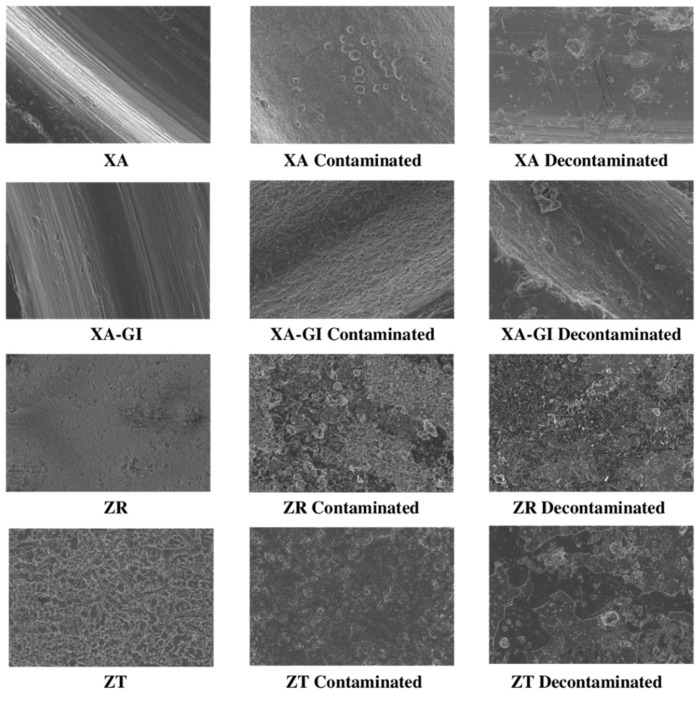
Micrographs of the sterile, contaminated by plaque and decontaminated XA, XA-GI, ZR and ZT surfaces.

SEM analysis confirmed the effectiveness of the decontamination process in removing the bacterial biofilm. After cleaning, the amount of residual organic material on the surfaces is generally very low, except for the ZR and ZT samples, which were found to have a high tendency for the retention of debris and biofilm residues. Crystalline structures (deriving from the sodium bicarbonate of the powder used) are commonly observed on cleaned surfaces; these formations typically measure a few microns but may constitute very extensive networks on some samples, such as ZT to ZR and RS surfaces. Electron microscopy investigations have also shown that the various types of discs react in very different ways to decontamination treatment regarding structural alteration. ZR and ZT samples show a profoundly affected surface with a complete loss of the original microtopography, which is also almost completely hidden by prominent residues and bacterial debris along with crystals, which are abundant in coverage and size. On the contrary, the treated surface of RS-GR5 disc shows only negligible damage, consisting in limited formation of minute pores (of 3–5 nm). Interesting differences in treatment response can be observed when comparing anodized samples with their counterparts that have not undergone electrochemical treatment. For example, the XA disc shows little surface alteration, whereas the corresponding XA-GI disc shows a very degraded surface, especially on the top of the ridges which appears chipped and with almost total loss of the anodized layer. The UTM sample shows severe scratches and flattening of the ridges, leading to an apparent narrowing of the groove width. Additionally, in the case of the respective anodized sample, the surface exhibits several damages in the form of indentations and irregularities, distributed both on the top and bottom of the ridges (Figure 2). In addition to these latter deteriorations, the almost complete loss of anodization can be observed (Figure 3 and Figure 4). Even in the RS disc samples, the treatment interferes with the preservation of the anodizing layer. However, except for the loss of this latter, the surfaces of both anodized and non-anodized samples do not appear to show any substantial changes to their micro-topographical features, except for the formation of minute pores, comparable in shape and size to those observed in the RS-GR5 samples. 

### 3.4. Statistical Analysis

If a correlation is investigated between roughness and wettability with respect to bacterial contamination, it is not possible to analyze the linear correlation, as the dataset is strongly non-homogeneous on a linear scale (the values are 0.2, 0.6, 1.2, 1.4, 21).

Instead, it is convenient to analyze the roughness on a logarithmic scale:

$${\mathrm{Rougness}}_{\log}=\mathrm{Roughness}$$

**Scheme 1:**
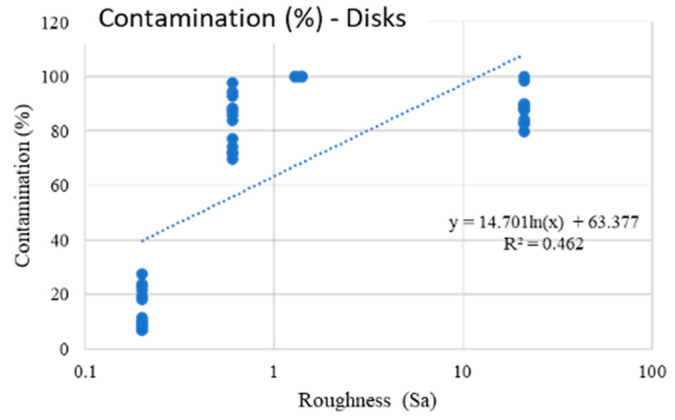
The present fit explains the linear correlation between the roughness on a logarithmic scale and the contamination of the disks.

If is calculated the linear correlation between the roughness on a logarithmic scale and the contamination is obtained the fit present in Scheme 1. However, the equation that best describes the roughness–contamination correlation is the following:

$$\mathrm{Contamination}=\left(1-e^{-a*\mathrm{Roughness}}\right)b+c$$

where:

a = 2.06 (CI 95%: 1.385, 2.735)

b = 2.931 (CI 95%: 0.349, 6.211)

c = 90.92 (CI 95%: 88.15, 93.96)

R^2^ = 95.59%; R^2^_adjusted_ = 95.41%

**Scheme 2:**
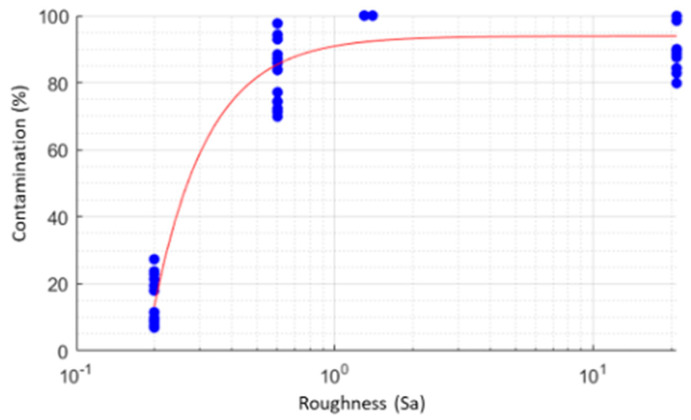
The present scheme indicates that the roughness has a very high influence on the bacterial contamination. When roughness increases over Sa 0.60, it is possible to observe a saturation process (values around 90–100% are reached).

The model represented in Scheme 2 indicates that the roughness has a very high influence on the contamination due to roughness less than 0.6, whereas as these increase there is a saturation process (also because values around 90–100% are reached).

Regarding the wettability, no relevant correlations are found (even using multivariate analyzes, it is always found that the only statistically significant variable (*p* = 0.05) is roughness).

If a correlation is sought between wettability and roughness with respect to the ability of a surface to be decontaminated with dust and ultrasound, it is possible to observe that the correlations are not significant.

**Scheme 3:**
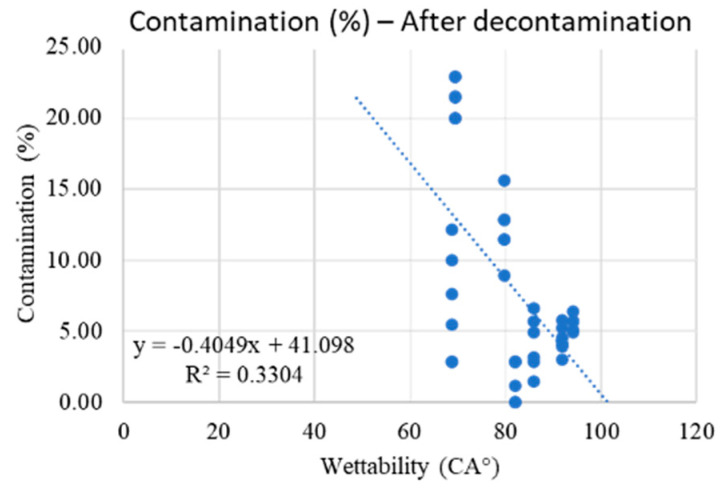
A “post-decontamination” surface model with linear trend is obtained if ZT and ZR are eliminated from the dataset.

However, if the ZT and ZR surfaces which are statistically highly anomalous, are eliminated from the dataset, is obtained a post-treatment surface contamination model with linear trend (Scheme 3).

The mathematical model to understand the contamination of remaining bacteria after decontamination was:

Contamination=a∗Roughness+b

where:

a = 2.863 (CI = 95%: 2.269, 3.457)

b = 7.916 (CI = 95%: 6.753, 9.079)

R^2^ = 68.86%.

R^2^_adjusted_ = 68.08%

The result shows that as the roughness increases, the number of bacteria that remain on the surface after decontamination treatments are higher and scale with a linear trend. However, the high variability of the data should be highlighted (which translates into an R2 that is not very high, but still significant).

ZT and ZR behave abnormally, as they remain highly contaminated (100%), even after treatment.

**Scheme 4:**
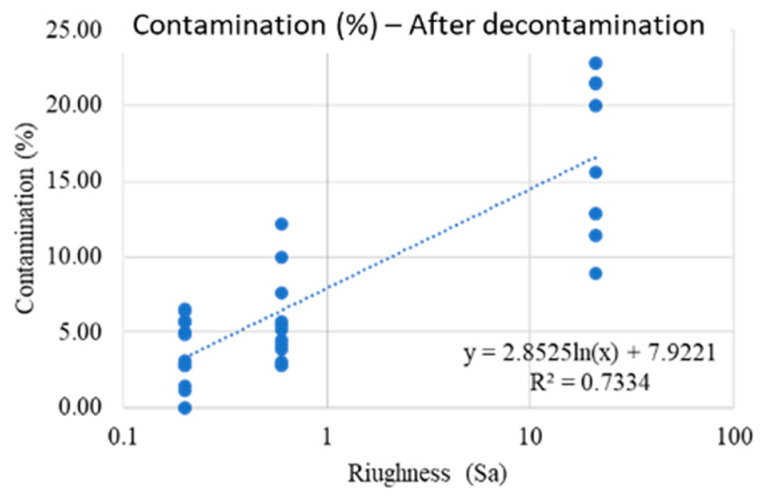
ZT and ZR surfaces remain highly contaminated (100%) even after treatment. If the ZT and ZR discs are excluded, there is a tendency of the material to decontaminate better in case of high wettability.

If the ZT and ZR discs are always excluded, there is a tendency for the material to decontaminate better in case of high wettability (Scheme 4). Therefore, the mathematical model able to relate wettability with respect to bacterial contamination after decontamination is the following:

Contamination=a∗Wettability+b

where:

a = −0.4049 (CI = 95%: −0.5892, −0.2207)

b = 41.1 (CI = 95%: 25.95, 56.24)

R^2^ = 33.04%.

R^2^_adjusted_ = 31.37%.

The “Roughness–Wettability” model was thus investigated and led to the following equation:

Contamination=a∗wettability+b∗roughness+c

where:

a = −0.142 (CI = 95%: −0.2712, −0.01475)

b = 0.5273 (CI = 95%: 0.4001, 0.6546)

c = 16.4 (CI = 95%: 5.461, 27.33)

R^2^ = 76.1%.

R^2^_adjusted_ = 74.87%.

The model indicates that both roughness and wettability play a role on the residual amount of contamination (excluding ZT and ZR).

It should be noted that the ZR and ZT surfaces have a contamination equal to 100% in all measurements, they show a clear tendency to become contaminated. Moreover, as already described, after the treatment the contamination remains, showing that these surfaces have a high resistance to decontamination.

Another anomalous behavior is given by RS GI, which has the same roughness as RS and RS GR5 (0.2), intermediate wettability but much higher contamination. By making a *t*-test for the difference of the means between RS, RS GR5 and RS GI, it is possible to obtain the results reported in Table 2 and Table 3.

There is a high contamination of RS GI that cannot be justified by the mathematical model.

The RG GR5 surface, on the other hand, tends to decontaminate more easily despite the lower wettability, with respect to RS and RS GI (Table 4 and Table 5).

**Scheme 5:**
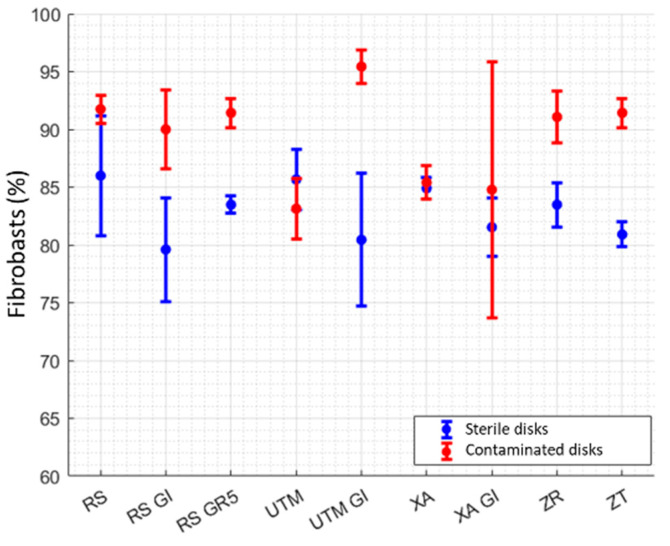
Percentages of fibroblasts on sterile and decontaminated discs.

**Figure 5:**
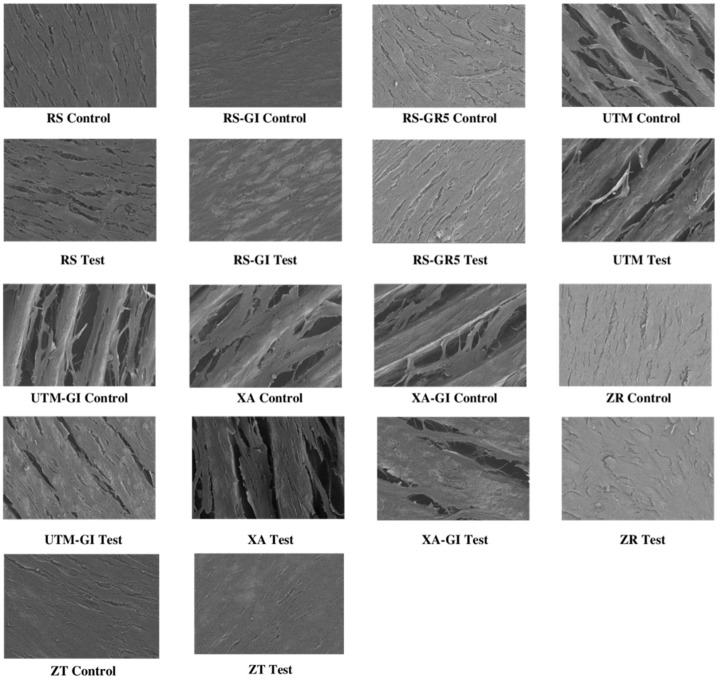
Micrographs of growth of fibroblast on the nine sterile (control) and decontaminated (test) surfaces.

Regarding the growth of fibroblasts, both in the case of sterile and decontaminated discs, the results are not statistically influenced by either roughness or wettability (Figure 5). In Scheme 5, it is possible to observe the percentages of fibroblasts on sterile and decontaminated discs. The results of the *t*-tests are shown in Table 6, with the surfaces with significant differences highlighted (alpha = 0.05).

## 4. Discussion

The increase in the number of implants inserted over the years has consequently led to an increasing demand for treatment of mucositis and peri-implantitis without the need for surgery.

The purpose of the following study was to understand from a microscopic point of view how nine different surfaces interact with the oral biofilm and how much the use of bicarbonate powders associated with ultrasonic instruments can decontaminate a surface. Finally, attention was paid to the growth of fibroblasts around these surfaces to understand the ability of these surfaces to allow fibroblastic growth after decontamination.

The SEM study of the nine surfaces has shown that roughness has a very high influence on contamination. Low contamination was shown when Sa was less than 0.6, whereas an increase in the Sa value results in a saturation process with a higher presence of bacteria. This agrees with a review of Dhaliwal et al., which [23] showed that the topographies have a beneficial effect on the bacterial colonization [24,25]. Bacterial colonization is reduced in machined titanium implants than in zirconia and other sandblasted and acid-etched surfaces [26,27]. SEM analysis has showed that ZT and ZR disks present a considerable quantity of small crevices and micro-porosities in which the presence of ecological bacterial niches became difficult to be decontaminated.

The wettability of a surface has been studied for a long time [28,29,30] because it allows the increase in surface energy with better adhesion of the integrins that allow the binding of mesenchymal cells in the early stages of the osseointegration process [31,32,33,34]. It could be thought that this bioactivity of the surfaces could also allow an easier bacterial colonization. The results of this study demonstrate that wettability cannot influence the surfaces susceptibility to increase biofilm formation. However, one of the limitations of the present study is the lack of data regarding bacterial adhesion and proliferation over a specific time frame. Some studies have hypothesized that this increased wettability may favor bacterial adhesion. Anyway, there is no consensus around this statement and a study of Schwarz et al. [35] concluded that hydrophilicity did not have an effect, whereas micro-topography had a highly and unpredictable influence on biofilm formation.

If the focus is shifted to capacity of powders and ultrasonic scaling to decontaminate the surfaces, this study has shown some interesting mathematical equations deriving from the metaregression analysis. If the ZT and ZR samples are removed from analysis due to their inability to be decontaminated, a slight correlation can be observed between wettability and plaque removement. This could be due to the possibility of creating a better contact between the particles of water sprayed by the airflow and the substrate to be decontaminated. However, no correlations were found in the literature prior to this study.

Many methods have been proposed in the literature for decontamination of implant surfaces such as the use of plastic and metal curettes, ultrasonic instruments, air-powder abrasive systems and titanium brushes [36].

In this study the samples were decontaminated using bicarbonate powder with high granulometry because in the literature it has been shown that bicarbonate powders guarantee a better holding of the surfaces than the others with smaller granulometry [13]. The use of ultrasound in this study was associated with the use of a stainless steel insert since it has been shown that the use of Teflon tips leads to the sedimentation of plastic particles on the surface nano-topography [37].

The results of metaregression have clearly shown that increasing the surface roughness causes a linear increase in the concentration of bacteria present along the surface.

On the other hand, studies have demonstrated that the use of bicarbonate powders and stainless steel tips has been shown to be effective in the treatment of biofilm, but they can cause damage to the surface topography [37,38].

The results of the SEM microscopic investigation have shown that the decontaminated surfaces always have a quantity of bicarbonate crystals present on the surface associated with changes in the micro-topography. In fact, the most substantial changes were observed on the samples in which there was an anodizing layer that has always been almost completely removed by the decontamination treatment. Another visible surface alteration was determined by the aggressiveness of the stainless-steel tip which caused scratches on the surfaces. Interestingly, the study seems to suggest that the choice of grade-5 Titanium alloys, being more resistant, is less susceptible to damage. The decontamination data also showed that a stronger alloy can allow for better decontamination. Some studies have pointed out how changing surface topography can affect hard and soft tissue cell proliferation [39]. For this reason, from a clinical point of view this would suggest preferring implants with grade 5 titanium alloys or Roxolid to allow better surface decontamination without altering the surface topography, especially when more abrasive instruments are used.

There is scientific evidence that particulate matter resulting from abrasion of instruments used and surfaces treated could lead to a foreign body reaction [40] in which activation of the cytokine cascade, metalloproteinases activation with subsequent inflammation can impair complete healing of the treated sites [41,42,43]. Certainly, other studies will also be needed to understand the immunohistochemistry of these decontaminated surfaces.

Regarding the growth of fibroblasts on surfaces, there are some interesting results. In fact, the surfaces RS, RS-GI, RS-GR5, UTM-GI, ZR and ZT show a statistically significant greater growth of fibroblasts in decontaminated surfaces compared with sterile surfaces.

This result can be interpreted in several ways and some speculations could be made. Primarily, the use of powders may have resulted in a surface nano-texture with roughnesses that favor the adhesion of the integrins that allow the adhesion of fibroblasts. Secondly, reference could be made to a recent study by Gianfreda et al. [33], showing that the presence of salts on implant surfaces (such as those of the powders used) determines a nano-roughness that increases the surface energy and makes it more wettable. In this way, even a smooth machined surface with a surface nanotexture could allow a significant response of the cells around it [43].

Interestingly, the anodized RS-GI and UTM-GI surfaces and the grade 5 titanium (RS-G5) surfaces after decontamination seem to favor the adhesion of fibroblasts. These results seem to agree with other studies which have shown that anodized surfaces are able to promote faster integration of peri-implant tissues. Similarly, the fibroblastic adhesion after decontamination appears to be significantly higher in the ZR and ZT surfaces which have a more articulated texture and richer in peaks and crests than the machined surfaces.

In this study it is emphasized that the growth of fibroblasts was carried out because it was interesting to observe what happens in the area of the implant-abutment junction.

Therefore, further studies will have to clarify whether this phenomenon concerning the greater growth of fibroblasts can also be translated to the growth of osteoblasts.

From a clinical point of view, certainly some precautions can be recommended following the results of the study. This study suggests to us primarily that it is not possible to obtain a total decontamination of the surfaces using exclusively powders and ultrasounds. If the abutment is removable in the presence of mucositis and peri-implantitis it would be advisable to remove it to decontaminate it and sterilize it extra-orally before reinserting it. Clinically, attention should be paid to the use of ultrasounds and powders because they alter the surface topography and there are no clear data in the literature regarding the growth performance of hard and soft tissue cells following these changes. Surely it would be more suitable to use more resistant alloys also to prevent the ultrasonic tips from damaging the implant connection when steel tips work on the implant head.

In addition, recent articles [24,25,44,45,46,47,48,49] showed the importance of different materials in the relation among abutment surface and soft tissue. It seems that there are no significant differences in terms of marginal bone level and bleeding on probing between machined and treated implant surfaces. This means that the abutments should always have smoother surfaces to allow better decontamination in cases of mucositis and peri-implantitis.

One of the limitations of the following study is the lack of fibroblast adhesion, proliferation and differentiation tests in the first moments after surface decontamination. Future studies will be needed to better understand the cell dynamics around these decontaminated surfaces.

## 5. Conclusions

The following study demonstrated how nine different types of surfaces used to produce implants and abutments react differently to bacterial colonization and decontamination with powders and ultrasounds. A negative correlation between roughness and the possibility of contamination has been demonstrated. The growth of fibroblasts gave discordant but statistically significant results. Future studies will be needed to fully understand how these decontaminated surfaces affect the adhesion, proliferation and differentiation of fibroblasts and osteoblasts.

## Figures and Tables

**Table 1:** Wettability and roughness of the different surfaces.

Surface	Contact Angle Analysis (°)	Roughness (Sa)
RS	94.2 ± 0.5	0.20
RS GI	85.9 ± 1.7	0.20
RS GR5	81.9 ± 1.3	0.20
UTM	68.8 ± 0.5	0.60
UTM GI	91.9 ± 1.3	0.60
XA	69.4 ± 1.7	21.00
XA GI	79.7 ± 4.6	21.00
ZT	114 ± 6.2	1.40
ZR	48.7 ± 2.7	1.20

**Table 2:** Contamination of RS, RS-GI and RS-GR5 expressed by mean and standard deviation.

Contamination	RS	RS GI	RS GR5
Mean	8.73	22.12	8.93
Standard Deviation	1.12	3.31	1.75

**Table 3:** Another anomalous behavior of RS-GI is confirmed by a *t*-test.

*p*-Value	RS	RS GI	RS GR5
RS	-	-	-
RS GI	3.69 × 10^−5^	-	-
RS GR5	0.41	1.75 × 10^−5^	-

**Table 4:** A high contamination of RS GI that cannot be justified by the mathematic model.

Bacterial Contamination after Decontamination	RS	RS GI	RS GR5
Mean	5.58	4.10	1.61
Standard Deviation	0.54	1.95	1.42

**Table 5:** The RG GR5 surface, on the other hand, tends to decontaminate more easily despite the lower wettability respect RS and RS GI. This could be due to higher strength of the alloy (Titanium Grade 5).

*p*-Value	RS	RS GI	RS GR5
RS	-	-	-
RS GI	0.06	-	-
RS GR5	2.58 × 10^−4^	0.02	-

**Table 6:** Percentages of fibroblasts on sterile and decontaminated discs. The results of the *t*-tests are with significant differences highlighted (alpha = 0.05).

	Sterile Disks		Decontaminated Disks		
	Mean	SD	Mean	SD	*p*-Value
RS	86.00	5.21	91.75	1.20	2.09%
RS GI	79.61	4.50	90.00	3.38	0.07%
RS GR5	83.48	0.74	91.43	1.28	0.00%
UTM	85.67	2.60	83.14	2.61	6.16%
UTM GI	80.44	5.76	95.44	1.47	0.05%
XA	84.90	0.92	85.41	1.47	24.29%
XA GI	81.54	2.56	84.78	11.07	25.62%
ZR	83.49	1.92	91.07	2.26	0.01%
ZT	80.91	1.09	91.43	1.28	0.00%

## Data Availability

The data presented in this study are available from the corresponding author upon request. The data are not publicly available due to privacy.
